# ANIPHI: An innovative pedagogical platform based on the Delphi method to support animal welfare teaching

**DOI:** 10.1371/journal.pone.0277189

**Published:** 2022-11-04

**Authors:** Ana C. L. Vieira, Michel Vidal, Jean-Baptiste Menassol, Teresa Letra Mateus, Ana Sofia Santos, Jean-Pierre Durieux, Mónica D. Oliveira

**Affiliations:** 1 Centre for Management Studies of Instituto Superior Técnico (CEG-IST), Universidade de Lisboa, Lisbon, Portugal; 2 Institut Agro Montpellier, Montpellier, France; 3 SELMET, Institut Agro Montpellier, CIRAD, INRAE, Univ Montpellier, Montpellier, France; 4 CISAS—Centre for Research and Development in Agrifood Systems and Sustainability, Escola Superior Agrária, Instituto Politécnico de Viana do Castelo, Rua Escola Industrial e Comercial Nun’Álvares, Viana do Castelo, Portugal; 5 Veterinary and Animal Research Centre (CECAV), UTAD, Associate Laboratory for Animal and Veterinary Sciences (AL4AnimalS) Quinta de Prados, Vila Real, Portugal; 6 EpiUnit–Instituto de Saúde Pública da Universidade do Porto, Porto, Portugal; 7 Ruralidade Verde, Lda, Qta dos Engenheiros, Vila Real, Portugal; 8 FeedInov CoLab, Estação Zootecnica Nacional, Qta da Fonte Boa, Vale de Santarém, Portugal; 9 S.E.N.S. Sciences Énergies Nature Santé, Esneux, Belgium; 10 iBB- Institute for Bioengineering and Biosciences and i4HB- Associate Laboratory Institute for Health and Bioeconomy, Instituto Superior Técnico, Universidade de Lisboa, Lisboa, Portugal; Universidade do Porto Instituto de Biologia Molecular e Celular, PORTUGAL

## Abstract

As a teaching subject, animal welfare is challenging for educators and learners, as was recently shown in a recent survey on the evolution of animal welfare teaching in Europe. Among several suggestions to overcome the current resistance to implementing animal welfare education, we highlight two. The first is that animal welfare education should be based on learner-centred approaches; the second is that it should encompass both animal welfare science and ethics and law. To the best of our knowledge, there are no learner-centred pedagogical approaches that can simultaneously explore scientific and ethical concepts. Furthermore, when exploring ethical concepts within the educational context, there is the additional challenge of being able to depart from discussion and debate to a systematic organization of knowledge. Our work simultaneously addresses these two challenges, presenting the design and implementation of a novel web-based learner-centred pedagogical platform for farm animal welfare teaching. The platform, named ANIPHI, uses the Delphi method’s iterative nature as a learning process to generate both reflection and (online) debate among learners. ANIPHI can be used by educators in an online environment, in a classroom environment, or in a combination of the two environments. ANIPHI was developed within the ERASMUS+ ANICARE project and is an open web-based platform for all educators interested in teaching farm animal welfare. Given ANIPHI’s flexible and user-friendly nature, the platform simultaneously exposes learners to ethical and scientific concepts in different educational realities, according to the educator’s objectives. Furthermore, videos depicting different husbandry practices across different types of animal production and countries are embedded in the platform. These videos are commented on by the farmer himself and by animal scientists, which enriches the learner’s experience. Educators across the ANICARE consortium have already successfully tested the ANIPHI platform for different farm animal welfare topics. We conclude this article by presenting one example of using ANIPHI in a real-life educational context, where we discuss some aspects of the design and use of our pedagogical platform.

## Introduction

As a teaching subject, farm animal welfare is challenging for educators and learners. When teaching farm animal welfare, educators need to be prepared to incorporate issues related to animal welfare science (e.g., how to measure animal welfare) and animal ethics and legislation (e.g., how we as humans should act regarding animals). When learning about farm animal welfare, learners must move on from their preconceptions around the subject and be open to consider scientific concepts and approaches, and also different perspectives. From the authors’ experience, there is usually much in-class controversy when approaching the subject. We can hypothesize that this, as in society, stems from “mixing up the scientific questions about the actual state of the welfare of animals and ethical questions about how we ought to treat and care for animals” ([1], p. 16). This controversy can be more or less enhanced depending on the background and training of the learners, as farm animal welfare is taught to different audiences that will have more or less scientific and empirical/experiential knowledge. For instance, for future farmers, loyalty and habits acquired concerning parental practices, conformity with the dominant practices, and ideologies of the professional world may create tension with scientific concepts, and there are psychological and cultural obstacles to take into account in introducing them [2].

These challenges can be even more enhanced depending on the educators’ ability (and/or experience) to conflate scientific and ethical concepts. A recent survey on the evolution of animal welfare teaching in Europe showed different points of resistance to implementing animal welfare education and suggested overcoming them through learner-centred approaches that can encompass animal welfare science with ethics and law [3]. Educators seem to prioritize animal welfare science concepts and methods favouring the learning of universal models (as the model of the five freedoms) [3]. It seems complicated for them to take advantage of critical pedagogical configurations (such as discussion and debate) that would allow them to explore ethics topics [2]. However, by avoiding fostering critical thinking, there is a missed opportunity to present to learners an integrated perspective that could enable them to apprehend scientific concepts while reasoning about animal ethics. In recent years, different pedagogical strategies have been proposed to deal with this encounter: debates and role-playing sessions [4], project-based learning and gamification [5], network learning [6]. Furthermore, multiple online resources based on technological platforms have also arisen, such as the computer-aided learning (CAL) packages on the topics of the welfare of husbandry systems [7] and small-animal husbandry [8], and the interactive learning tool “Animal Ethics Dilemma” [9]. These strategies and resources aim to promote more collaborative learning around the subject. Nevertheless, to the best of our knowledge, there are no pedagogical approaches that can simultaneously explore scientific and ethical concepts. Furthermore, when exploring ethical concepts within this context, there is the additional challenge of being able to depart from discussion and debate to a systematic organization of knowledge. This is the two-fold innovation of our work.

Our proposal in this article is to consider farm animal welfare teaching under the realm of Socially Acute Question (SAQ) [10, 11], whose pedagogical stakes we propose to consider. SAQ, as part of the socio-constructivist educational paradigm [10, 11], aims at the emancipation of the learner through educational practices that promote the development of critical, care and creative thinking. In particular, it studies the use of (face-to-face) debates as a training tool with three objectives: education for democratic life, the ability to debate, and learning knowledge on socio-ethical issues [12]. The SAQ-like debate aims to build a reflection in discourse and argumentation, to build shared understanding and promote consensus. Beyond the challenges of scientific debate, it mobilizes interdisciplinary and evolving reference knowledge, value systems, and socio-ethical or political choices [11, 12]. Still, SAQ-like debates can be time-consuming and therefore have to be considered according to the duration of the educational course. Furthermore, not all learners (or educators) feel comfortable presenting and discussing arguments in front of colleagues (sometimes of other cultural backgrounds), particularly when exploring acute social questions, such as farm animal welfare. Finally, as already mentioned, educators have the additional challenge of conducting a systematic organization of the knowledge and information collected within the debates so that the learners can understand and assimilate it, as with scientific knowledge.

The main objective of this article is to present the design and implementation of a novel web-based pedagogical platform to overcome the challenges presented when implementing SAQ-like debates as part of animal welfare teaching. The platform, named ANIPHI, uses the Delphi method’s iterative nature [13] as a learning process to generate both reflection and (online) debate among learners. ANIPHI can be used by educators in a totally online environment (where learners access the platform from other locations than the classroom), in a classroom environment (for example, in informatic labs where learners access ANIPHI individually) or in a combination of the two environments. ANIPHI was developed within the ERASMUS+ ANICARE (ERASMUS + 2017-1-FR01-KA202-037287) project and is an open web-based platform available for all educators interested in teaching farm animal welfare.

The following sections of the article are organized as follows. The next section presents the rationale for the ANIPHI platform design, providing user (educator perspective) interface guidance on how to use the platform to support SAQ-like debates. The following section explains ‘The ANIPHI platform architecture organization’. After that, we present one example of using ANIPHI in a real-life educational context. This example enables us to discuss some aspects of the design and use of our pedagogical platform in real education settings. Finally, in the last section, we provide the main conclusions by comparing ANIPHI with other teaching practices and tools in the field.

## The ANIPHI platform design

Delphi, a “method for structuring a group communication process so that the process is effective in allowing a group of individuals, as a whole, to deal with a complex problem” ([13], p.3), was selected as the conceptual background for the development of the ANIPHI platform. Delphi is recognized as a valuable ‘knowledge acquisition technique’ for developing different models of knowledge [14, 15]. Despite this, its use for educational purposes has been scant, with only a few references being found in the literature (see, for example, [16–18]). Nevertheless, it is our understanding that the Delphi’s four key features [19] make the method suitable for educational purposes, and particularly for overcoming the challenges presented when implementing SAQ-like debates in a class environment. Namely, (i) being anonymous (or quasi-anonymous) allows learners to provide their opinions freely, (ii) being iterative allows and promotes reflection, (iii) having controlled feedback exposes participants to different views, and (iv) having statistical aggregation allows participants to consider a large volume of information.

Given the rise of the use of technological platforms, there has been a sharp increase in the last years in the use of the online or web-Delphi. The web-Delphi retains all the Delphi method standard features while simultaneously taking advantage of advances in technological platforms. Hence, the web-Delphi type allows to include audio and visual stimuli, which meets the objectives of proving debate triggers and, ultimately, promoting learners’ engagement. Finally, we cannot circumvent the fact that technology has become part of learners and educators alike daily life [6, 20–22] and has also been recognized as a “groundbreaking advancement in education” ([18], p. 464).

**Fig 1:**
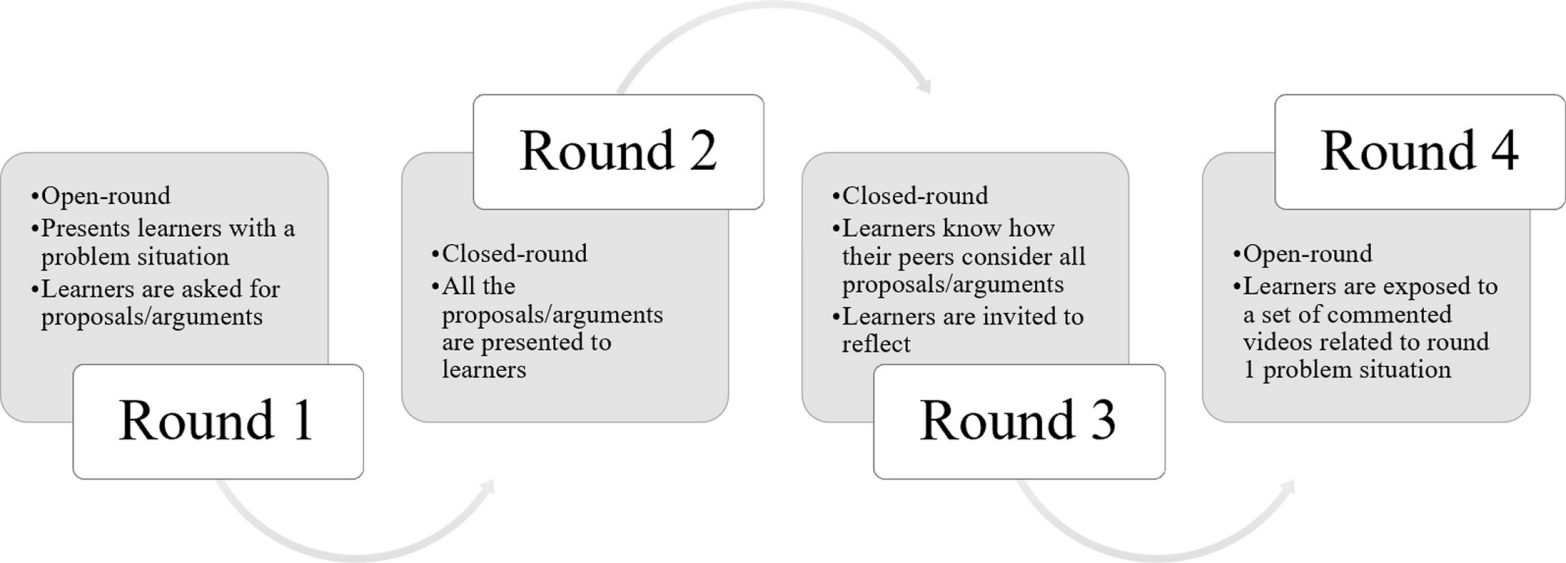
Schematic presentation of the design of the ANIPHI template. The figure details the specific round requests and challenges.

For ANIPHI, our methodological option was to design a template for a Delphi process with four rounds with specific requests and challenges to learners (for a brief description, see **Fig 1**). The learners access each ANIPHI round using individual confidential access that is sent to him/her through the platform by the educators, and that can be used within the classroom or from other locations. Each learner will have to register at the platform by creating an individual password-protected account.

The first round is an open round where learners are confronted with a text prepared by educators (that can be complemented with images) that describes a problem situation. In this first round, learners are asked one question as the trigger question for debate (**Fig 1**). At the end of the first round, the educators scan through the learners’ answers and set a list of proposals/arguments. This will be the input for the second round (**Fig 1**). The second and third rounds are closed rounds. The difference between the two is that, in the second round, all the proposals/arguments are presented to learners in an equal stance–free from any agreement, or disagreement, considerations; distinctively, in the third round, learners get acquainted with how their peers consider the different proposals/arguments that have been put forward. In both rounds, learners can ‘keep silent’ by answering ‘Don’t know/Don’t want to answer’, as they would have in a class environment, and provide comments individually for each proposal/argument, as well as general comments about the process itself. The feature to leave comments is critical in our design, as the rationale behind learners’ opinions around the topic is as important as their answers. Furthermore, the general comments are also instrumental in allowing educators to pick up ‘the feeling of the room’ regarding the debate process being conducted. Finally, in the fourth round, learners are exposed to a set of commented videos selected to have a connection with the initial situation presented in the first round, and, with them, we aim to expose learners to new perspectives and experiences around the problem situation (**Fig 1**). We conclude the fourth round with two questions that aim to understand what learners have learned about the problem situation, from other learners’ considerations, and what their opinion was regarding the pedagogical approach and their own situation within the class. We advocate that the use of the ANIPHI platform should be framed as preparatory work and warm-up for face-to-face debate, and that is up to the educator to decide when face-to-face exchanges are to take place.

Operationally, learners can only participate in the processes to which they are invited. Still, they will be invited to each round, irrespective of their participation in the previous rounds, as in real-life educational contexts, a learner can miss a class and continue their engagement in the course. The educators control the collaborative environment by managing the ANIPHI platform, where each process is created, employing a set of features organized in an architecture described in the next section. It is important to understand that the group of learners enrolled in a Delphi process can, in practice, overcome the limits of the classroom, as it is feasible having two or more different groups of learners engaged in the same ANIPHI process, being the process facilitated in co-share by two, or more, educators. Also, each educator can run multiple and even simultaneous processes with different groups of learners. There are no limits of participants to be included as learners in an ANIPHI process, other than the limitations imposed by the increased burden of processing all the answers in the first round and comments made by participants. It is also important to highlight that both the learners and the educators can use the platform by themselves, with no need for support from IT services, as both only need their individual computers with online access from which they can reach the ANIPHI platform. Nevertheless, there is always the possibility for direct exchanges with ANIPHI developers with the mailbox support@welphi.com, and access to tutorials (that can be found here https://erasmus-anicare.eu/?AniPhi) that were created for educators so that they can implement ANIPHI through a hands-on approach.

ANIPHI was developed in collaboration with Decision Eyes, a Portuguese Start-up responsible for the development of WELPHI, a software solution on top of which ANIPHI was developed (for further information, see http://www.welphi.com/). Decision Eyes will keep assisting the authors in maintaining the database, fixing bugs, and modifying the platform if needed. Educators using ANIPHI can analyze, delete and export data collected in all rounds. All the information collected is stored following WELPHI’s Privacy Policy which can be found at https://app.welphi.com/welphi/TermsAndConditions/Anicare/TermsEN.pdf. The platform is available in the English, French, Portuguese and Spanish languages at http://app.welphi.com/welphidev/Pages/LoginPage.aspx.

In the next section, we present the ANIPHI platform architecture organization so that educators can understand how to implement the ANIPHI design and provide user interface guidance on how to use the platform to support SAQ-like debates.

## The ANIPHI platform architecture organization

The ANIPHI platform is a free-to-use web-based application developed using the .NET framework connected to an SQL Server 2014 database and accessible through the Internet. In terms of its architecture, ANIPHI is organized into 14 pages: (1) create login, (2) process overview, (3) round details management (round 1 and 4), (4) round details management (round 2 and 3), (5) process details management, (6) edit and manage round 1 info, (7) edit and manage round 2 and 3 info, (8) edit and manage round 4 info, (9) edit messages, (10) add and manage proposals/arguments, (11) add and manage participants, (12) add and manage scale level, (13) proposals/arguments statistics, and (14) participants statistics. In this section, we will present screenshots of ANIPHI to illustrate the platform architecture organization: selected screenshots reference the case study presented in the next section. For this reason, the reader will see the platform in French (one of the four languages in which ANIPHI is available, while the problem situation, questions and arguments are all in English, the language of the exercise conducted).

**Fig 2:**
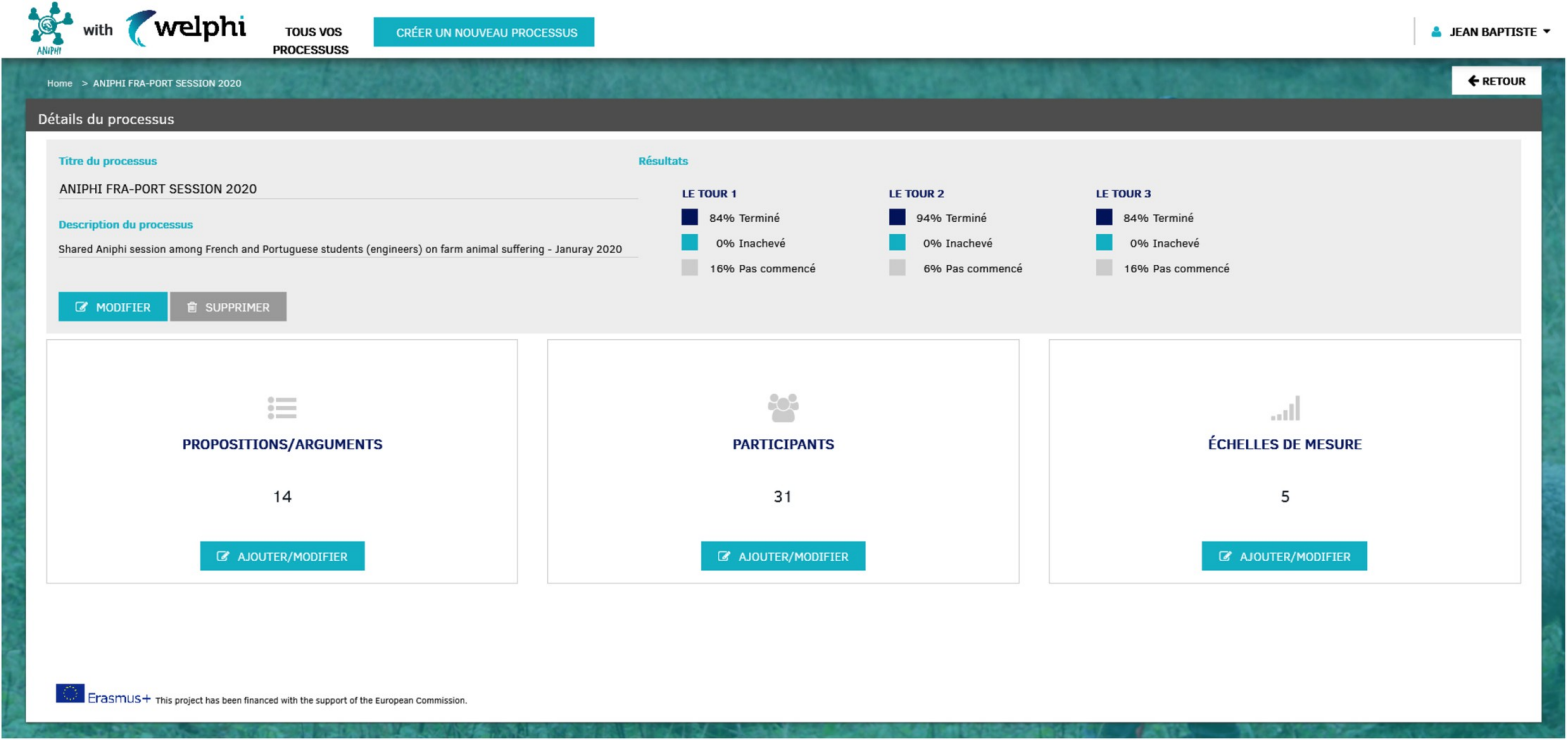
Process details management (user: Educator).

When starting a new process in ANIPHI, the educators are directed to the ‘process details management’ page (**Fig 2**) where they will find three sections leading to three individual pages: ‘add and manage proposals/arguments’, ‘add and manage participants’ and ‘add and manage scale level’. Before the first round is concluded, only the ‘Participants’ section is editable. Following round 1, the educators are then able to add and edit the ‘proposals/arguments’ and ‘scale levels’ sections.

**Fig 3:**
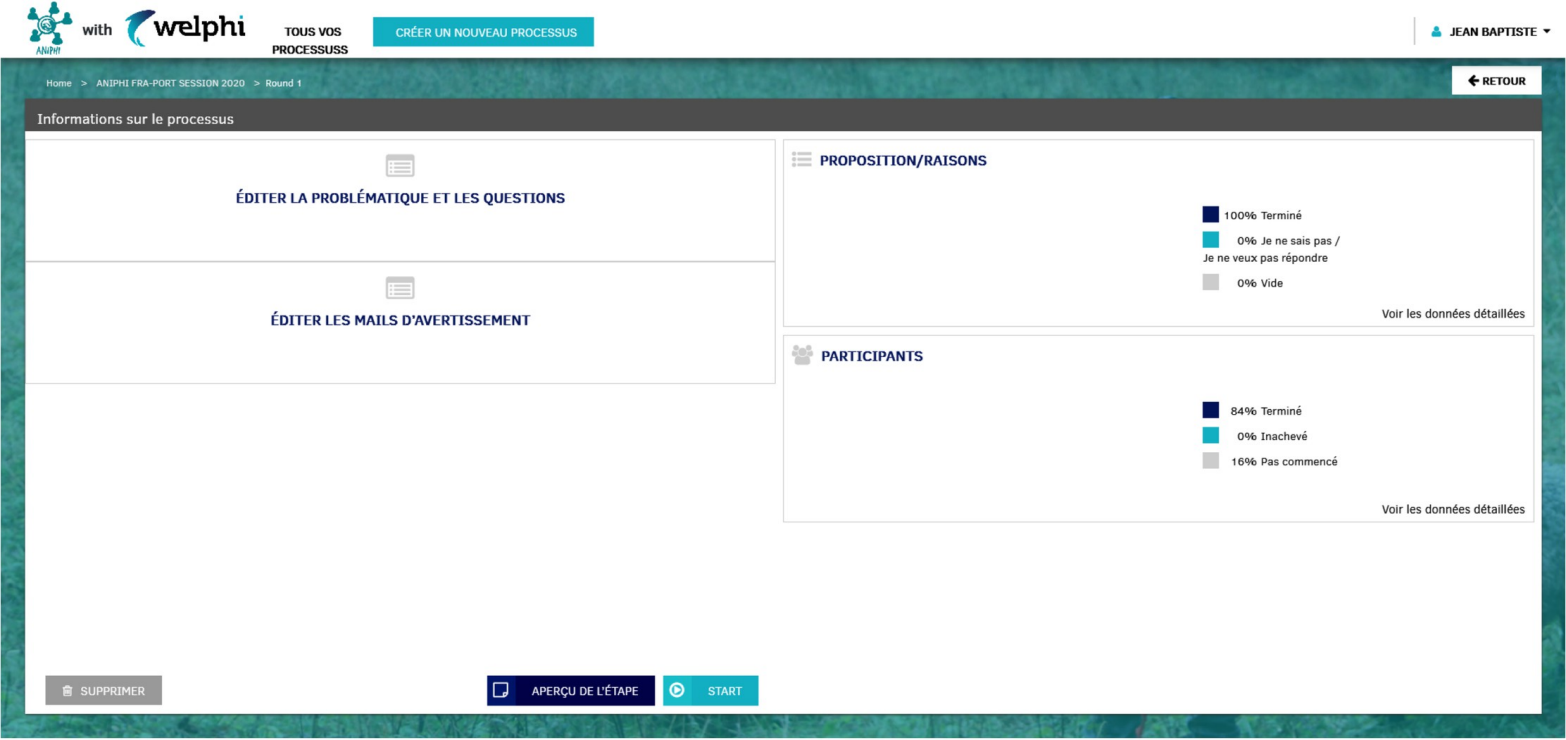
Round details management, for rounds 1 and 4 (user: Educator).

**Fig 3** represents the ‘Round details management’ page for round 1 (that will later be the same for round 4). The ‘round details management’ page is divided into four sections. The ‘Edit round template’ section is located in the upper left area of the screen, where the description of the problem situation is to be presented to the learners, and the question is inserted. The ‘Edit rounds and warning emails messages’ section is located in the down left area of the screen, and is where the messages used to communicate with the participants during the ANIPHI process are edited. There are two types of messages. The ‘Welcome message’ and the ‘Thank you message’ appear on the round’s questionnaire; the other messages are e-mail messages that will be used to interact with the participants. Each message already has a template text that educators can edit. The ‘Proposals/arguments’ section is located in the upper right of the screen, where the participants’ answers are analyzed. Finally, the ‘Participants’ section is located in the lower right area of the screen, and it is where educators interact with the participants using the messages above described.

An additional feature present in the ‘Round details management’ is the ‘PREVIEW ROUND’ button, which shows the educators a replica of what participants will see, hence being possible to validate the design of the first round. When educators are ready to start, they click the ‘START’ button, and when the deadline established for the first round is reached, or when all the participants have answered, they can close the round and analyze the answers by clicking the ‘CLOSE’ button.

**Fig 4:**
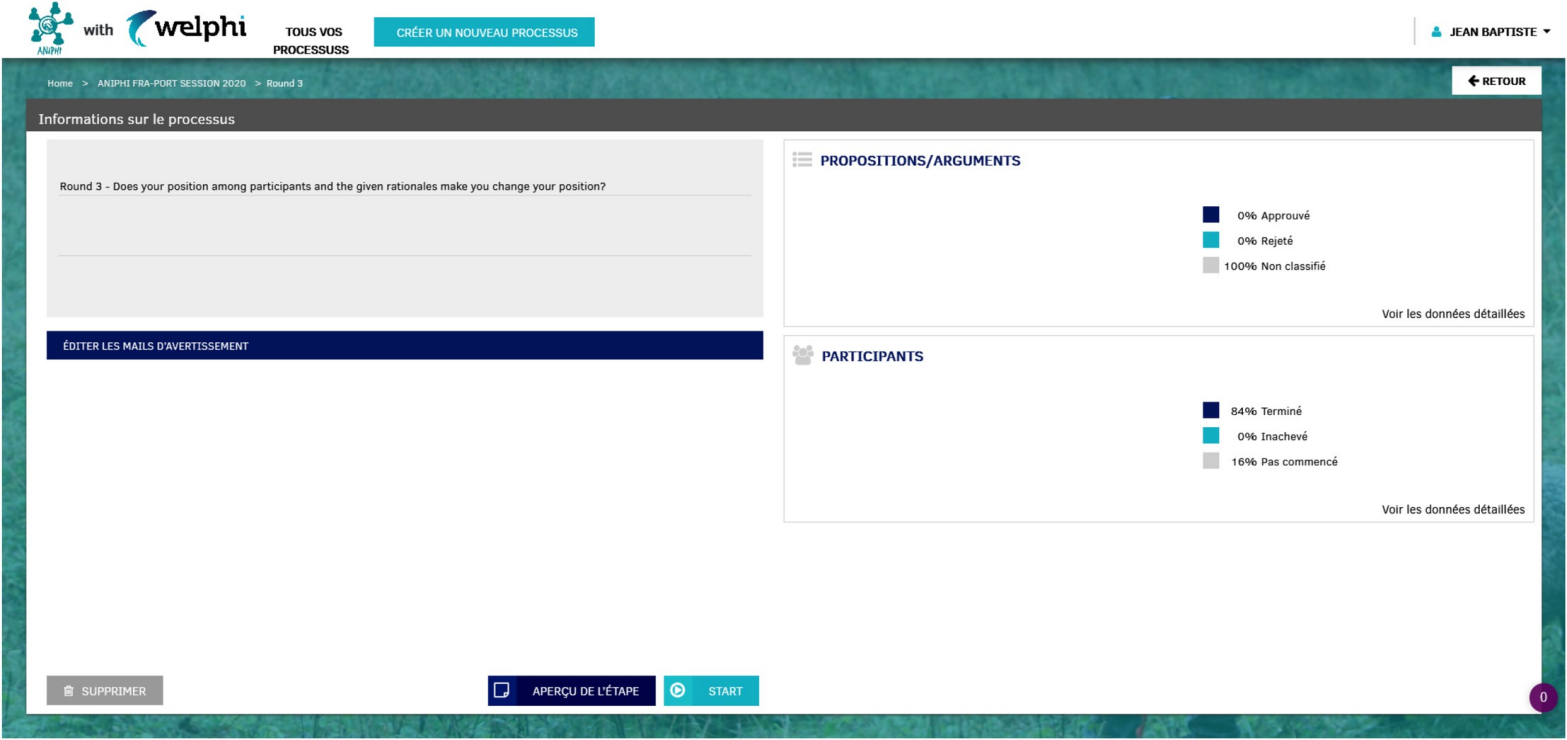
Round details management, for round 2 and 3 (user: Educator).

**Fig 4** represents the ‘Round details management (for Round 2 and 3)’ page where the educators can edit the questions to be presented in both rounds, and, after the round starts, can monitor the learners’ participation. The structure and features of the page are very similar to what was already described for the ‘Round details management (for Round 1 and 4)’ page (**Fig 3**).

**Fig 5:**
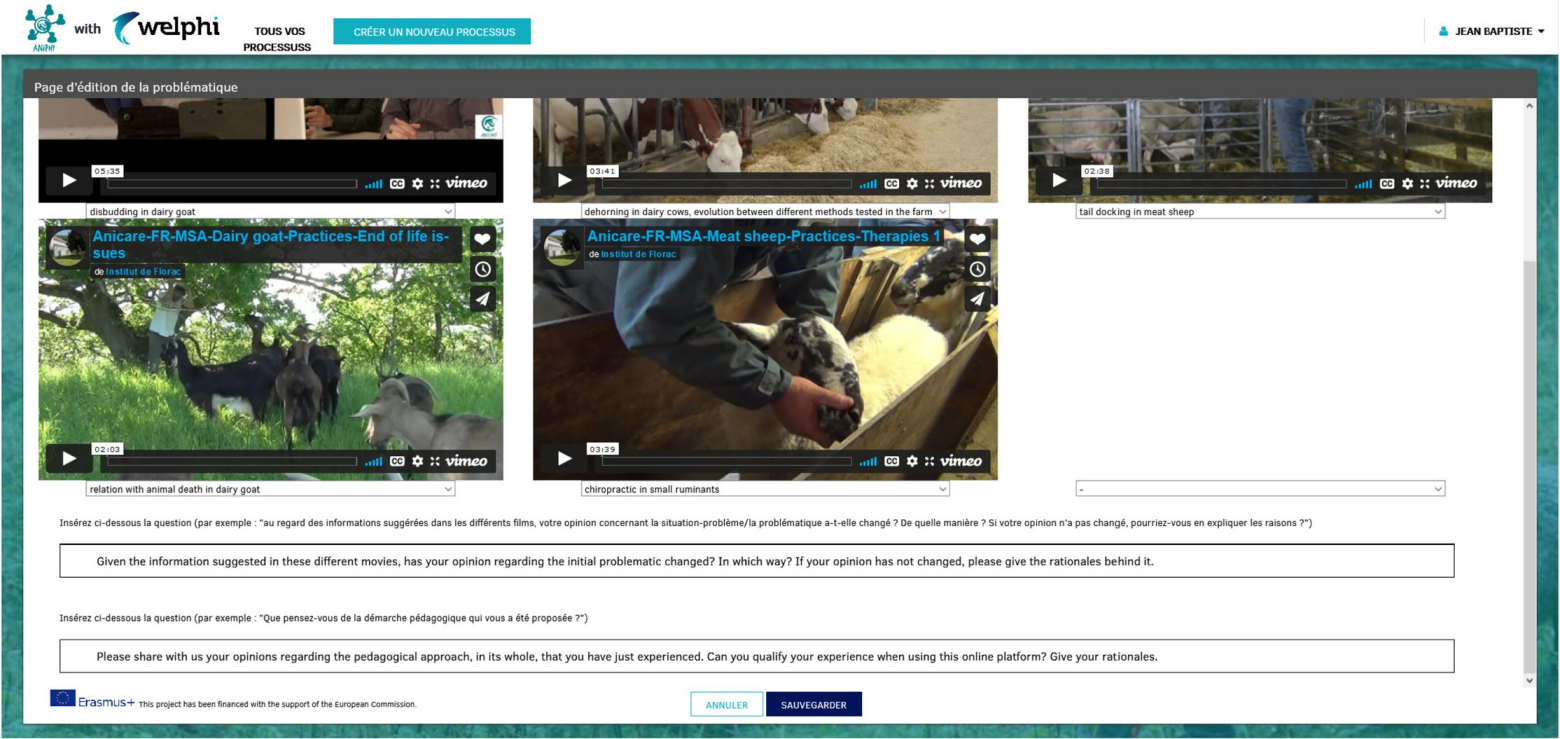
Edit and manage round 4 information.

**Fig 5** presents the ‘Edit and manage round 4 info’ page where the educators can select the set of videos he/she wants the learners to see and edit the questions to be presented to learners. The educators can choose from the ANICARE project pool of videos found at https://erasmus-anicare.eu/?MainPage, and add from one up to six videos. There are different types of videos, namely videos of farmer practices commented by the farmer itself or by another ‘expert’. Our proposal is to have two questions in this last round: one to understand what learners have learned about the problem situation, and another to collect their opinion regarding the pedagogical approach itself.

## Using ANIPHI in real-life educational contexts

Within the ANICARE consortium, the ANIPHI platform has been used in several educational contexts and with distinct types of learners. From these in-class tests, we observe that it is crucial to adapt the initial problem situation to the type of learners and to the specific learning objectives. For instance, technical students (training to obtain an Advanced Agricultural Technician Certificate) felt comfortable participating in pedagogical exercises where they worked on on-farm practical problems (for example, an adjustment in the nutritional program or bedding conditions); whereas veterinary students participated with enthusiasm on clinical and welfare challenges. Comparatively, agronomy students were more likely to integrate higher farm components to propose a multi-scale analysis. Initial problem situations that corresponded to an ethical dilemma were able to satisfy all types of educational backgrounds, both during online (irrespective of where they were taking place) and in face-to-face exchanges. This section presents a case study on one educational context in which we have used ANIPHI to confront learners with an ethical dilemma. ANIPHI was used in a combination between an online environment (where learners access the platform from other locations than the classroom), and within a classroom environment. In the description of the case study, we focus mainly on the results of using the platform and provide some insights from the educators/facilitators.

### Case study—Breeding conditions and practices that cause suffering in farm animals

We developed and implemented an ethical dilemma exercise involving 31 engineering learners, 21 from the French engineering school *L’Institut Agro–Montpellier SupAgro* (Montpellier, France) and ten from the Portuguese University *Universidade de Trás-os-Montes e Alto Douro* (UTAD–Vila Real, Portugal). Different cultural backgrounds were privileged to multiply the representations, values and arguments around the same problem. The main goal of this exercise was to approach the general topic: *‘*breeding conditions and practices that cause suffering in farm animals’. The exercise took place in March 2019, lasted 12 days and was composed of face-to-face exchanges (intra-site) and by-distance exchanges (inter-site), briefly presented in the following sub-sections. We used the ANIPHI platform as the tool for inter-site exchanges, where we aimed to develop critical and creative thinking skills on the topic under analysis. The objective of the intra-site exchanges was to determine the beliefs and feelings of the learners regarding the topic, evaluate the effects of the inter-site exchanges, and provide learners with the available body of scientific knowledge that learners of farm animal welfare need to acquire.

#### First intra-site exchange: Introducing the ethical dilemma

The first intra-site exchange was inspired by the Cooperative Learning in Multicultural groups (CLIM) method (which lasted around three hours). It took place in both Montpellier and UTAD. It consisted of a sequence of intra-group (5–6 learners) and inter-group exchanges supported by different types of pedagogical materials and methods: free talks regarding the general topic, a Q-sort to sort out 20 given affirmations regarding a given question ‘Some situations faced by farm animals can negatively impact their welfare: is it problematic?’, the suggestion of reading and summarising a pool of relevant technical and scientific publications, and a request of a plenary presentation of a general topic in relation with farm animal welfare (e.g. ‘A cooperative of pig breeders invites you to present the need to improve farm animal welfare in order to reduce suffering for these animals. On which aspects do you choose to focus your attention?’). These first exchanges enabled the learners to debate animal welfare conceptions and be confronted with technical and scientific concepts regarding farm animal welfare. For the generality of the learners, this first stage was helpful in getting involved in the topic and enabled them to consider the following by-distance exchanges with better attention regarding the differences in cultural representations and the controversies at stake.

#### By-distance exchanges (inter-sites): Using the ANIPHI platform

Following a brief presentation of the ANIPHI platform, learners were given access to the platform for seven consecutive days. The learners completed the first three rounds individually as personal homework during extra-curricular times, while round 4 was completed individually during a face-to-face session.

The initial problem situation and question presented at the beginning of **round 1** were the following:

‘In Europe, we can now size up the effects of 30 years of legislation and regulation regarding farm animal protection and farm animal welfare. Nevertheless, despite these efforts, some situations still trigger suffering in farm animals. According to your own experience, knowledge and values, why do you think this is so? How would you recommend ending these occurrences of suffering?’

At the end of round 1, the learners’ answers were summarised in a list of arguments (proposals and recommendations) by the educator/facilitator. During **round 2**, that list was published, and each student was allowed to provide her/his level of agreement, or disagreement, toward each item, using a five-level Likert-type scale (Strongly agree, Agree, Neither agree nor disagree, Disagree, Strongly disagree). While doing so, the educators strongly recommended that learners give the rationales behind their opinions. During **round 3**, and considering the information collected in round 2, the learners were once again confronted with the same list of ideas and were given the option to keep or change their initial opinions given the opinions of the other learners. Again, the educators strongly encouraged the learners to provide the rationale for their decision. Answers in English were favoured, but students were left free to answer in their native language if they felt they could not express their rationale in English. The list of four proposals and ten recommendations given by the learners can be found in S1 Table (together with the final results collected in round 3 and presented in S2 Table), and are the example of how, through the iterative nature of Delphi, it is possible in this context to being able to depart from discussion and debate to a systematic organization of knowledge.

The final results of the Delphi process show that some learners changed their initial opinion expressed in round 2; however, these changes were of low amplitude ranging from 1 to 5 per cent, i.e. one or two learners changing their opinion for each item. Different types of behaviour could be observed, with some learners firmly standing and putting forward their answers and rationales, while others more likely to compare their answers and rationales with other learners. The most agreed proposal was Proposal 2 ‘Urban population & anthropocentrism’, while Proposal 3 ‘Rich country preoccupation’ and Proposal 4 ‘One Welfare’ were strongly debated. The most agreed recommendations were Recommendation 1 ‘Adapt the system to the animal’, Recommendation 2 ‘Legislation, research and control’ and Recommendation 3 ‘Education from a young age’. The recommendations most debated were Recommendation 4 ‘Consumers & European label for Animal Welfare’ and Recommendation 9 ‘Reduce meat consumption’. Proposals with many contributors were often consensual among the group except for Recommendation 4, with a significant number of contradictors. Only learners of the French school made proposals in an attempt to explain the situation, while the learners of the Portuguese university only proposed recommendations. By a vast majority, the Portuguese university learners’ recommendations were connected with legislation and rule enforcement topics (8 / 9).

**Round 4** of the process was chosen by educators to be a live classroom session (one in each participating country). It was based upon a selection of four short movies made during the ANICARE project and related to the general topic of the pedagogical approach, in particular, ‘dehorning in dairy cow’, ‘chiropractic in small ruminants’, ‘tail docking in meat sheep’, ‘disbudding in dairy goats’, ‘relation with animal death in dairy goats’. Learners watched the videos together, but the round exercise was completed individually using their access to the platform. Finally, educator and learners conducted a post-assessment of the process itself. Completing the by-distance exchanges using the ANIPHI platform was not mandatory for the learners since this training was not graded. Nevertheless, among the 31 learners originally invited, only two learners did not participate. Hence, 84% (26 / 31 learners) participated in rounds 1 and 94% (29 / 31 learners) in rounds 2 and 3.

#### Final intra-site exchange: Concluding the ethical dilemma exercise

The final intra-site exchanges (one in each participating country) lasted around 60 minutes and focused on the learnings brought by being exposed to different arguments and the movies and their possible influences on the learners’ answers and/or comments during the inter-site exchanges. The learners considered it essential to observe concrete professional practices to enable them to go beyond an overly academic vision of the problem situation, re-analyze it through farmers’ empirical knowledge, and discover practices they did not know, thus extending their knowledge. Nevertheless, they also considered that they did not have sufficient technical and scientific knowledge on some of the topics and expressed the urgency to move towards a more scientific exploration.

#### Learners’ and educators’ feedback

After the first intra-site exchange, five volunteer learners (only in the French school) participated in a focus group to conduct a self-reflection about the pedagogical approach. The discussion was recorded. The questions focused on the actions of the learner during the process, her/his feeling, values, intentions, and type of knowledge involved. The interviews with the same learners were repeated at the end of the last intra-site exchange. The following topics were explored: (1) practical point of view in using the ANIPHI platform (e.g. time spent, number of accesses, instructions), (2) how did the students deal with other opinions, (3) how did they feel in making an informed and independent choice, (4) did they feel their opinion was important for the other learners involved in the process, and how did they perceive their contribution to the process. All learners confirmed that the pedagogical approach allowed them to express their opinions freely and therefore without fear of judgment and of being subjected to intrusive positioning and/or pressure from other learners. Most importantly, their motivation toward the pedagogical approach gradually increased. Three stages were particularly stimulating for them: (a) first, seeing the arguments/ideas they had stated in round 1 appear at the beginning of round 2. The interviewees felt they were being listened to and respected because the educators did not judge them. During this same stage, learning new arguments aroused critical reflection rather than questioning their points of view. (b) second, at the beginning of round 3, when they heard the opinions of the other participants concerning the arguments they had put forward. Reading them stimulated their desire to react in order to counter-argue. (c) third, during round 4, they discovered new practices that led them to support or complement their initial point of view. This last round triggered questions and doubts and enabled them to connect with their farming experience. However, the learners also expressed some technical difficulties during their engagement in the process (e.g. good Internet connection). Furthermore, some of the students considered the rhythm of the process too slow, particularly the long waiting time between each round. Nevertheless, when questioned about possible solutions, students were conscious that a faster implementation time would not have been better, as time was needed for reflection.

In terms of the pedagogical experience from the educator’s point of view, some situations were found to be critical. When students did not recognize their arguments in the list of those produced at the end of round 1 (because the argument was either misunderstood or incorrectly rephrased by the educator), this was likely to generate a feeling of non-recognition in the learner. The inability of each participant to react to comments and arguments directed at him/her can also be a source of frustration. The time spent on each round was also one of the most challenging issues for the educator to manage, as the process should allow sufficient time for each individual to integrate what has been learned but not too much time to make it fastidious. The exploration of the results in rounds 2 and 3 can be time-consuming, and adding automatically-generated graphs could help improve the learning experience. Finally, technical problems (inability to connect to the platform, to record their answers) can lead learners to abandon the process. For all these reasons, it is vital for educators to carefully select and set the environment in which ANIPHI is used and always combine it with face-to-face exchanges.

## Discussion

This article proposes a new open web-based pedagogical platform named ANIPHI that is freely available for all educators interested in teaching farm animal welfare and is offered in the English, French, Portuguese, and Spanish languages. Traditional educational practices regarding farm animal welfare are centred on lecture-based classes with a top-bottom conception of knowledge (both theoretical and practical) transfer [4]. This platform responds to a previously identified need for a greater diversity in the pedagogical design and environment of teachings in animal welfare that could complete these traditional forms [6]. Amongst existing pedagogical alternatives, ANIPHI presents a proven advantage in the sense that it allows filling a gap in terms of (i) learners’ integration, (ii) learning objectives and (iii) learning environment.

Regarding learners’ integration, innovative methodologies are developed in many institutions that promote learner-centred teaching practices [23, 24] but often do not benefit from dedicated tools and/or have a vertical knowledge transfer approach. A common feature of these animal welfare educational practices is directed toward one specific kind of learner (student in veterinary or agronomy sciences, educators) [7, 8]. Innovative media such as Massive Open Online Courses (MOOC) can tackle this limitation [6, 25]. However, the core of traditional practices appears to be unchanged in these courses, as exchanges between participants are merely a side benefit of the platform features, mainly used between learners and not capitalized as a production of the course itself. Moreover, when innovative teaching methods emphasizing collaborative learning exist [26], they appear to be limited to on-site exchanges and debates. Furthermore, when courses are explicitly developed for the operators of the livestock sector (farmers, lorry drivers, …), they have mainly focused on technical and commercial aspects of farm animal welfare [23] and lack tools to assess the quality of the educational program itself as well as its impacts on the learners [24]. ANIPHI answers these challenges, allowing learners from distinct backgrounds (and eventually from different countries) to make structured debates.

Regarding learning objectives, ANIPHI does not focus on disseminating and implementing scientific knowledge about animal behaviour and animal needs [6, 7], nor on animal ethics and legislation. Thus, this pedagogical platform does not come with a criterion-referenced evaluation of the knowledge and skills related to animal welfare; in this respect, it is similar to another existing learning tool called “Animal Ethics Dilemma” [9]. However, compared with the latter, the design of ANIPHI does not target an autonomous and individual training experience. Rather, its learning objectives are centred around the development of critical, care and creative thinking of the learners within a collective and collaborative process. These transversal skills targeted by ANIPHI are intimately associated with the debate sequences (formalization, expression and sharing of one’s point of view, the identification of the various positions in the debate, the understanding of the rationales behind other’s positions, …) and, therefore, constitute strong originality of this platform within the current landscape of available tools and methods to teach animal welfare.

Finally, and in comparison with the previously cited learning tools or methods, the learning environment of ANIPHI is remarkably adaptable, with teaching methods combining face-to-face and by-distance learning sequences in various proportions between both. Indeed, this pedagogical platform was not solely designed as a by-distance learning tool for learners but offers the possibility to be integrated within a broader pedagogical approach that integrates one or more educators. ANIPHI achieves its intended purpose in the sense that it allows overcoming the challenges presented when implementing SAQ-like debates in a class environment, being an innovative tool to equip educators that may feel, in some contexts, more comfortable with implementing this type of teaching with their learners. ANIPHI allows for a more democratic teaching format where the educators do not assume the role of experts, and in which all learners have a voice. In view of the above, the uniqueness of ANIPHI does not allow for it to be compared with other methods and tools as a way of validation. Instead, we argue that it constitutes a complementary and essential pedagogical tool to teach farm animal welfare to the broadest range of learners in various pedagogical environments.

## Main conclusions and future work

The ANIPHI platform is available to be used within a collaborative learning setting, and its main strength is the promotion of a dialogue between values and experiential and scientific knowledge to develop critical, care and creative thinking. ANIPHI is a free, intuitive, and easy-to-use educational platform, as experience from pre-tests and the presented case study show. We argue that by using ANIPHI learners will feel more comfortable and prepared to participate in the debates in class. Educators will also feel more in control to lead such a debate and work with the learners. Complemented with tutorials and instructions for users, it is very accessible to educators that wish to implement SAQ-like debates in a class environment and complement it with other educational formats. Furthermore, it is aligned with new ways of education and training being explored in the 21st century, complementing in-class and online teaching; and helps educators from different countries promote joint discussions. ANIPHI is being continuously monitored for improvement, and we hope in future to make it accessible for other educators looking into other SAQ (e.g. sustainability, technological assessment and adoption in health contexts).

## Supporting information

S1 Table The list of four proposals and ten recommendations. (DOCX)

S2 Table Results of round 3 extracted from the ANIPHI platform: Distribution of the level of agreement/disagreement for each item of the list built from the ideas collected during round 1. (DOCX)
