# Supplementary material for: ANIPHI: An innovative pedagogical platform based on the Delphi method to support animal welfare teaching
Source: PLoS One. 2022 Nov 4;17(11):e0277189. doi: 10.1371/journal.pone.0277189 (PMC9635751; doi:10.1371/journal.pone.0277189)
Supplement: S1 Table — (DOCX) [file pone.0277189.s001.docx]

# **Supporting information. Additional information regarding the by-distance exchanges (inter-sites): using the ANIPHI platform**

**S1 Table. The list of four proposals and ten recommendations.**

| **Proposal 1** | The intensification of the livestock sector and mass production of meat are largely responsible for this situation". Industrial-scale production is the problem (8 F). |
| --- | --- |
| **Proposal 2** | Urban population, who take a great place in society debates, has seen a great decrease in its link with the agricultural sector. These populations do not understand farm practices and tend to interpret some common and normal situations as situations that trigger animal suffering (by anthropocentrism or when comparing with their pets). In this sense, regulation through legislation can be problematic since laws are made by politicians from Brussel and are often not suited for specific working conditions which can be the best for the farmer and the animals". Urban population & anthropocentrism (4 F). |
| **Proposal 3** | Depending on socio-economic factors, the need for animal products (to reduce hunger) can be stronger than preoccupations about animal welfare". Rich country preoccupation (1 F). |
| **Proposal 4** | These situations that generate suffering often originate from a complicated personal situation of the breeder (low income, stress, …) which leads the breeder not to be able to take good care of the animals". One Welfare (1 F). |
|  | |
| **Recommendation 1** | The production systems need to be more adapted to the animals (and not the contrary), their own intelligence, perceptions and sensitivity. This kind of systems will develop a positive aspect of the work of farmers for them and consumers. In the same manner, the slaughterhouses should be designed to embrace animal perceptions (olfaction, visual perceptions…) and their behaviour (social needs…) to reduce pain". Adapt the system to the animal (10 F). |
| **Recommendation 2** | An improvement of the actual legislation and new laws based on scientific studies and facts could help in reducing animal suffering along with more practical evaluation and controls. In addition, legislation could be improved by using a shared European database constructed from on-farm audits (funded by the European common agricultural policy) to help in identifying problematic situations". Legislation, research and control (5 FR – 3 P). |
| **Recommendation 3** | The education of people from a young age, integrating humans as part of a global ecosystem and valuing trough communication the knowledge and practices of farmers is a good way to improve animal welfare in a collective manner". Education from a young age (6 F – 1 P). |
| **Recommendation 4** | Consumers will be the main actor to induce a change in the way we treat farm animal by deciding which products to consume or not and in favour of production systems that are favourable to animal welfare. There should be a generalization of a European label that guarantee shared values for good animal welfare to make consumers more sensitive to animal suffering when buying animal products and that would guarantee a better selling price for farmers". Consumers & European label for Animal Welfare (3 F – 2P). |
| **Recommendation 5** | Working conditions in industrial slaughter houses are not compatible with the rules regarding animal welfare, reduction in volumes and on-farm slaughter should be enhanced in order to reduce the transport of living animals and thereby extending a good animal welfare management all along the life cycle of the animal". Reduce volumes, transports and rhythm in industrial slaughterhouses (4 F – 1 P). |
| **Recommendation 6** | Reduction in animal suffering and improvement of farm animal welfare depends on the breeder and the associated breeding system: the quality of the human-animal relationship, the personal investments in satisfying animal needs, their technical knowledge and performances. Therefore, we should promote more peasant farming since it improves the human-animal relationship and satisfy consumers demands for humane treatments and traceability. It will also help to put more trust in our breeders". Peasant farming & Human-Animal Relationships (5 F). |
| **Recommendation 7** | There is no "miracle" solution to reduce animal suffering. This will be achieved by collective decision-making among people from the livestock sector and citizen representatives to improve breeding conditions of farm animals while maintaining this activity economically-profitable". Collective decision-making (4 F). |
| **Recommendation 8** | The investments in welfare education, evaluation and controls are not sufficient enough to ensure the application of the right measures and use of appropriate material to reduce suffering. Moreover, the application of legal sanctions (financial fines…) are not coercive enough to fully force large companies to apply laws that would reduce animal suffering; there should be enforcement of legal provisions". More investments to promote and punish (1 F – 2P). |
| **Recommendation 9** | Reduction in meat consumption is an ideal solution to achieve a quantitative lowering in animal suffering as well as a potential greater qualitative investment to reduce situations that provoke suffering". Reduce meat consumption (2 F). |
| **Recommendation 10** | Paying the right price for animal products and thereby excluding financial subsidies, is a solution to improve animal welfare by valuing the work of the farmers". Paying the right price (1 F). |
