# Supplementary material for: ANIPHI: An innovative pedagogical platform based on the Delphi method to support animal welfare teaching
Source: PLoS One. 2022 Nov 4;17(11):e0277189. doi: 10.1371/journal.pone.0277189 (PMC9635751; doi:10.1371/journal.pone.0277189)
Supplement: S2 Table — (DOCX) [file pone.0277189.s002.docx]

# **Supporting information. Additional information regarding the by-distance exchanges (inter-sites): using the ANIPHI platform**

**S2 Table. Results of round 3 extracted from the ANIPHI platform: distribution of the level of agreement/disagreement for each item of the list built from the ideas collected during round 1.**

|  | Strongly agree | Agree | Neither agree nor disagree | Disagree | Strongly disagree |
| --- | --- | --- | --- | --- | --- |
| Proposal 1 | 31% | 54% | 8% | 8% |  |
| Proposal 2 | 50% | 31% | 15% | 4% |  |
| Proposal 3 | 19% | 12% | 19% | 38% | 12% |
| Proposal 4 | 8% | 46% | 19% | 8% | 19% |
| Recommendation 1 | 65% | 23% | 12% |  |  |
| Recommendation 2 | 35% | 58% | 8% |  |  |
| Recommendation 3 | 73% | 27% |  |  |  |
| Recommendation 4 | 38% | 15% | 31% | 12% | 4% |
| Recommendation 5 | 35% | 35% | 19% | 12% |  |
| Recommendation 6 | 42% | 46% | 8% | 4% |  |
| Recommendation 7 | 35% | 50% | 15% |  |  |
| Recommendation 8 | 19% | 42% | 35% |  | 4% |
| Recommendation 9 | 15% | 38% | 19% | 19% | 8% |
| Recommendation 10 | 19% | 58% | 15% | 4% | 4% |
